# Shedding Light on Autophagy During Human Tuberculosis. A Long Way to Go

**DOI:** 10.3389/fcimb.2021.820095

**Published:** 2022-01-05

**Authors:** Joaquin Miguel Pellegrini, Nancy Liliana Tateosian, María Paula Morelli, Verónica Edith García

**Affiliations:** ^1^ Centre d’Immunologie de Marseille-Luminy, Aix Marseille Université, INSERM, CNRS, Marseille, France; ^2^ Departamento de Química Biológica, Facultad de Ciencias Exactas y Naturales, Universidad de Buenos Aires, Buenos Aires, Argentina; ^3^ Instituto de Química Biológica de la Facultad de Ciencias Exactas y Naturales (IQUIBICEN), Facultad de Ciencias Exactas y Naturales, Universidad de Buenos Aires, Consejo Nacional de Investigaciones Científicas y Técnicas, Buenos Aires, Argentina

**Keywords:** autophagy, tuberculosis, human, host-directed therapy, immunology & infectious diseases

## Abstract

Immunity against *Mycobacterium tuberculosis* (*Mtb*) is highly complex, and the outcome of the infection depends on the role of several immune mediators with particular temporal dynamics on the host microenvironment. Autophagy is a central homeostatic mechanism that plays a role on immunity against intracellular pathogens, including *Mtb*. Enhanced autophagy in macrophages mediates elimination of intracellular *Mtb* through lytic and antimicrobial properties only found in autolysosomes. Additionally, it has been demonstrated that standard anti-tuberculosis chemotherapy depends on host autophagy to coordinate successful antimicrobial responses to mycobacteria. Notably, autophagy constitutes an anti-inflammatory mechanism that protects against endomembrane damage triggered by several endogenous components or infectious agents and precludes excessive inflammation. It has also been reported that autophagy can be modulated by cytokines and other immunological signals. Most of the studies on autophagy as a defense mechanism against *Mycobacterium* have been performed using murine models or human cell lines. However, very limited information exists about the autophagic response in cells from tuberculosis patients. Herein, we review studies that face the autophagy process in tuberculosis patients as a component of the immune response of the human host against an intracellular microorganism such as *Mtb*. Interestingly, these findings might contribute to recognize new targets for the development of novel therapeutic tools to combat *Mtb*. Actually, either as a potential successful vaccine or a complementary immunotherapy, efforts are needed to further elucidate the role of autophagy during the immune response of the human host, which will allow to achieve protective and therapeutic benefits in human tuberculosis.

## Introduction

*Mycobacterium tuberculosis* (*Mtb*) has killed nearly 1000 million people since the XIX century. And although an affordable and effective treatment is available to fight this pathogen, tuberculosis (TB), together with COVID19 in 2020-2021, is the leading cause of death from a single infectious agent. Therefore, improvement of treatment is included among the central aims of developing new strategies against this disease. Accordingly, it has been proposed that supplementing anti-TB therapy with host response modulators will augment standard TB treatment (Madhur et al., 2016). However, the immune response against *Mtb* is highly complex. The outcome of TB infection depends, at least in part, on several immune mediators that display critical temporal roles on the host microenvironment (Sodhi et al., 1997; Ottenhoff et al., 1998; Chen et al., 2008; Pasquinelli et al., 2009; Cooper, 2010; Jurado et al., 2012; Mayer-Barber et al., 2014; Pellegrini et al., 2021). It has been suggested that host-directed therapies (HDT) could be untapped strategies as complementary therapies against TB, augmenting the host defences and/or limiting tissue damage associated with infection (Martínez-Colón and Moore, 2018; Wan et al., 2018; Xiong et al., 2018). In this context, autophagy arises as an attractive therapeutic target, but currently available data on autophagy in TB patients and the potential clinical use of this cellular process remain insufficient. Here, we review the current knowledge of autophagy as a potential complement of anti-TB chemotherapy.

## Autophagy

Autophagy is an evolutionarily-conserved cellular process that mediates the lysosomal degradation of cytoplasmic components and damaged organelles, allowing eukaryotic cells to generate nutrients under starvation conditions and maintain cellular homeostasis. Three types of autophagy have been described: chaperone-mediated autophagy, microautophagy, and macroautophagy, herein referred to as autophagy (Jacomin et al., 2018). Importantly, autophagy participates in innate and adaptive immunity against intracellular pathogens, including *Mtb* (Gutierrez et al., 2004). Actually, increased autophagy in macrophages eliminates intracellular *Mtb via* lytic and antimicrobial mechanisms of the autolysosomes (Ponpuak et al., 2010). Notably, autophagy constitutes an anti-inflammatory mechanism that protects against endomembrane damage triggered by several endogenous components or infectious agents and precludes excessive inflammation (Castillo et al., 2012; Deretic and Levine, 2018). The autophagy process can be modulated by different immunological mediators (Djavaheri-Mergny et al., 2006). In particular, critical cytokines regulate both positively and negatively the autophagic response affecting survival of mycobacteria (Harris et al., 2007). Besides, the importance of the host autophagy process to manage an effective antimicrobial effect on mycobacteria during chemotherapy has been reported (Kim et al., 2012). A better understanding of the connections between autophagy and the immune response may have wide applications given that the pathology accompanying several diseases involves some form of inflammation (Deretic and Levine, 2018).

## Autophagy in TB Patients

Most of the studies that investigated autophagy as a defense mechanism against *Mtb* have been accomplished in murine cell lines, mouse models, primary culture cells, or human cell lines infected with the pathogen. However, very limited information regarding the study of the autophagic response in TB patients is available. During the past decade, we have studied *Mtb*-induced autophagy in two populations of patients with active disease, classified according to their T cell responses to the bacterium. Briefly, high responder (HR) TB patients displayed significant T cell proliferation and IFN-γ production against *Mtb-*antigen (Ag), while low responder (LR) TB patients displayed weak or no T cell responses to the antigen (Pasquinelli et al., 2004). Interestingly, we detected the highest autophagy levels in healthy donor (HD)´s monocytes whereas the lowest quantities were observed in monocytes from LR patients (Rovetta et al., 2014). Accordingly, it has been reported that Beclin-1, a signaling hub of autophagy, is increased in alveolar macrophages from TB patients and that those individuals with higher Beclin-1 levels achieve faster bacillary sterilization (Yu et al., 2016). Recently, we observed that autophagy levels decreased significantly in neutrophils from TB patients as compared to HD (Pellegrini et al., 2020). Moreover, a direct correlation between neutrophil numbers and TB severity was detected (Pellegrini et al., 2020). Given that during *Mtb* infection autophagy protects against massive inflammation (Deretic and Levine, 2018), the reduced levels of autophagy observed in neutrophils from TB patients could be related to the frequent harmful inflammatory responses that take place during active disease.

### Effect of the Diversity of *Mtb* Strains on the Autophagy Process

The immune response to *Mtb* is influenced by factors both from the host and the bacteria (Sousa et al., 2020). Accordingly, some studies have demonstrated a differential ability of different *Mtb* strains to modulate autophagy. In particular, Li et al. described that clinical isolates from *Mtb* differ in their ability to induce autophagosome formation (Li et al., 2016). The authors investigated the effect of more than 180 *Mtb* clinical isolates on the autophagy process in THP-1 macrophages. Interestingly, they observed that the capacity of inducing autophagy varied significantly among different isolates. Notably, patients infected with *Mtb* strains that displayed reduced autophagy-inducing ability showed more severe disease and displayed adverse treatment outcomes, suggesting that an autophagy deficiency induced by *Mtb* isolates augmented the risk of poor clinical outcomes in TB patients (Li et al., 2016).

The majority of the studies on the host immune response to *Mtb* infection have been performed using the laboratory strain H37Rv (see **Tables 1**–**3**). Moreover, most of the research performed with samples from TB patients does not consider the original *Mtb* strain that infected the host. Thus, investigation of the effect of *Mtb* genetic variability on the modulation of the autophagy process is of great interest.

**Table 1:** Immunological mediators modulate the autophagy process during active tuberculosis.

Immunological mediators	Effect on autophagy	Validation in human samples	*Mtb strain*	Host origin	Reference
TNF	Stimulation	Osteoarticular pathological tissues from TB patients. Validated with osteoclasts from HD	*Mtb* H37Rv and H37RvΔeis	Chinese men cohort from Wuhan	Liu G. et al. (2020)
IFN-γ	Stimulation	Monocyte-derived macrophages from HD	*M. bovis* BCG	Not detailed	Gutierrez et al. (2004)
		Monocytes from TB patients and HD	*Mtb* H37Rv whole cell lysate	Argentine population cohort from Buenos Aires	Rovetta et al. (2014)
		Monocytes from TB patients and HD	*Mtb* H37Rv, *Mtb* H37RvΔRD1 and *Mtb* H37Rv whole cell lysate	Argentine population cohort from Buenos Aires	Tateosian et al. (2017)
IL-4, IL-10, IL-13	Inhibition	Human cell lines U937 and THP-1; Monocyte-derived macrophages	*M. bovis BCG*	Not detailed	Harris et al. (2007)
IL-17A	Stimulation	Monocytes from TB patients and HD	*Mtb* H37Rv, *Mtb* H37RvΔRD1 and *Mtb* H37Rv whole cell lysate	Argentine population cohort from Buenos Aires	Tateosian et al. (2017)
IL-26	Stimulation	Monocyte-derived macrophages from HD	*Mtb* H37Ra and *M. leprae*	Not detailed	Dang et al. (2019)
SLAMF1	Stimulation	Neutrophils from TB patients and HD	*Mtb* H37Rv whole cell lysate	Argentine population cohort from Buenos Aires (Caucasian, American Indian, Asian)	Pellegrini et al. (2020)
PGE2	Stimulation	Monocytes and neutrophils from TB patients and HD	*Mtb* H37Rv whole cell lysate	Argentine population cohort from Buenos Aires (Caucasian, American Indian, Asian)	Pellegrini et al. (2021)

A summary of published studies on the modulation of autophagy by different immunological mediators such as cytokines, glycoproteins and lipid compounds with validation in human samples is shown. Mtb strains employed and host origin are detailed.

**Table 2:** Non-coding RNAs influence autophagy outcome during human tuberculosis.

ncRNA	Target	Effect on autophagy	Validation in human samples	*Mtb* strain	Host origin	Reference
miR-30A	Beclin-1	Inhibition	Expression in alveolar macrophages, association with clinical data and treatment	*Mtb* H37Rv (*in vitro* functional experiments)	Chinese cohort from Beijing	Chen Z. et al. (2015)
miR144*	DRAM2	Inhibition	Expression in PBMCs and lung and lymph nodes biopsies from TB patients, functional experiments in human MDMs	*Mtb* H37Rv (*in vitro* functional experiments) miR144* expression confirmation upon infection with *Mtb* H37Ra, *M. bovi*s BCG and *M. abscessus*	Samples from Korea Biobank Network	Kim et al. (2017)
miR-125b-5p	DRAM2	not described	Expression in primary monocytes from TB patients	*Mtb* H37Rv	Chinese cohort from Xinjiang	Liu G. et al. (2020)
CircAGFG1	miRNA1257 - Notch	Stimulation	Expression and correlation with autophagy/apoptosis in alveolar macrophages	Not detailed	Chinese cohort from Chilin	Shi et al. (2020)
miRNA-27a	Cacna2d3	Inhibition	miRNA expression profiles from PBMCs of patients with active pulmonary TB	*Mtb* H37Rv	Chinese cohort from Shanghai	Liu et al. (2018)*
lncRNA-EPS	–	Inhibition	Negative correlation with LC3 levels in monocytes from TB patients	–	Chinese cohort from Wuhan	Ke et al. (2020)**
PCED1B-AS1	miR-155	Stimulation	Expression in peripheral monocytes from TB patients, functional experiments in human MDMs	*Mtb* H37Rv (*in vitro* functional experiments)	Chinese cohort from Xinxiang	Li et al. (2019)
miR-155	ATG3	Inhibition	Expression and functional experiments in *Mtb*-infected human dendritic cells	*Mtb* H37Rv	Samples from Blood Bank of University "La Sapienza", Italy	Etna et al. (2018)
miRNA-889	TWEAK	Inhibition	miRNA next-generation sequencing (NGS) analysis in PBMC of RA patients with latent TB infection, functional experiments in human PBMCs	*Mtb* H37Rv and *M. bovis* BCG (*in vitro* functional experiments)	Taiwanes cohort from Taichung	Chen et al. (2020)

A summary of published studies on the modulation of autophagy by non-coding RNAs with validation in human samples is shown. Mtb strains employed and host origin are detailed. ^*^DOI: 10.1038/s41467-018-06836-4; ^**^DOI: 10.1016/j.meegid.2019.104077.

**Table 3:** SNPs in autophagy-related genes associated with TB. List of SNPs in genes codifying for proteins involved in autophagy that have been found to be associated with increasing or decreasing susceptibility to active TB.

Gene	SNP	Alleles	Consequence	Association with TB	*Mtb* strain involved	Host origin	Reference
**IRGM1**	rs9637876	C>T	Non Coding Transcript Variant	Decreased susceptibility	*Mtb* Euro-American lineage (and not TB caused by by *M. africanum* or *M. bovis*)	Patients cohort from Ghana (Ahsanti, Eastern and Central regions)	Intemann et al. (2009)
**IRGM1**	rs10065172	C>A,T	Missense Variant	Increased susceptibility among African Americans	Not determined	Caucasian and African American patients cohort from Boston, EEUU,	King et al. (2011)
**IRGM1**	rs10065172 rs10051924 rs13361189	C>A,T T>A,C T>C	Missense Variant Non Coding Transcript Variant	Increased susceptibility	Chinese patients cohort from Hubei Han region	Not determined	Lu et al. (2016)
**IRGM1**	rs4958846	T>C	2KB Upstream Variant	Decreased susceptibility	Not determined	Chinese patients cohort from Hubei Han region	Yuan et al. (2016)
**ULK1**	rs12297124 rs7300908	G>T C>T	Intron Variant Intron Variant	Associated with latent TB	Not determined	Patients cohort from Seattle, EEUU, self-identified as black or Asian	Horne et al. (2016)
**LAMP1**	rs9577229	C>T	Missense Variant	Increased susceptibility	*Mtb* Beijing genotype	Indonesian patients cohort from Jakarta and Bandung regions	Songane et al. (2012)
**MTOR**	rs6701524	A>G	Intron Variant	Increased susceptibility	*Mtb* Beijing genotype	Indonesian patients cohort from Jakarta and Bandung regions	Songane et al. (2012)
**P2X7**	SNP000063002 (−762)	C>T	762b Upstream Variant	Decreased susceptibility	Not determined	Patients cohort from the western region of Gambia	Li et al. (2002)
**P2X7**	1513A-C	A>C	Missense Variant	Increased susceptibility	Not determined	Cohorts of refugee and australian patients with northern european and vietnamese ancestry	Fernando et al. (2007)
**VAMP8**	rs1010	T>C / T>G	3 Prime UTR Variant	Increased susceptibility	Not determined	Chinese patients cohort from Hubei Han region	Cheng et al. (2019)

Mtb strain corresponds to the specific strains or clinical isolates that originally infected the host.

Genome sequence analysis has identified seven phylogeographic *Mtb* lineages: four referred to as evolutionarily “ancient” and three as “modern”. Interestingly, the “modern” strains were shown to display high virulence (Romagnoli et al., 2018). Therefore, Romagnoli et al. investigated the impact of the genetic diversity of *Mtb* strains on the host innate immune response by evaluating the autophagy response. Remarkably, the authors demonstrated that modern *Mtb* strains are able to avoid the autophagy machinery affecting the regulation of specific T-cell responses.

Together, the studies described above might suggest a possible limitation of using autophagy as a novel therapy against *Mtb*. However, on the other side, it was proposed that analyses of autophagosome formation by diverse clinical isolates of *Mtb* might contribute to the evaluation of TB outcomes (Li et al., 2016). Furthermore, the study of the genetic variability of *Mtb* on autophagy modulation was proposed to have translational implications for the design of HDT, which should consider both the autophagic and immunogenic properties of the lineage of the *Mtb* candidate. Accordingly, by studying 681 TB patients Sousa et al. showed that *Mtb* isolates from cases with mild disease stimulate strong cytokine responses in contrast to bacteria from patients with severe TB, indicating that *Mtb* strains manipulate host-pathogen interactions to drive variable TB severities. Then, they suggest to include *Mtb* genetic diversity in the development of HDT (Sousa et al., 2020). Finally, external autophagy modulators might act as adjuvants in *Mtb* treatment helping to overcome autophagy regulation/inhibition by pathogenic strains.

### Immunological Mediators

Autophagy is a process recognized to be regulated by cytokines and other immunological signals (Djavaheri-Mergny et al., 2006; Harris et al., 2007; Goletti et al., 2013; Chen H. et al., 2015; Pelissier-Rota et al., 2015; Martínez-Colón and Moore, 2018; Wan et al., 2018). TNF was originally shown to induce autophagy in Ewing sarcoma cells (Djavaheri-Mergny et al., 2006). Recently, TNF was demonstrated to promote the autophagy of *Mtb*-infected osteoclasts and constrain the apoptosis of mature osteoclasts (Liu W. et al., 2020). Furthermore, Liu et al. suggest that their data describe a novel osteoarticular TB-activated cytokine network where autophagy could have an important role in the pathogenesis of osteoarticular TB, pointing out the use of drugs such as TNF for treating this type of TB (Liu W. et al., 2020). Moreover, IFN-γ augments the autophagy process in macrophages and other cells (Gutierrez et al., 2004; Goletti et al., 2013), whereas IL-4, IL-10 and IL-13 inhibited autophagy in murine macrophages and human cell lines (Harris et al., 2007; Park et al., 2011). Accordingly, it has been demonstrated that autophagy participates in the immune response of TB patients against *Mtb*, in direct association with the specific IFN-γ levels secreted against the pathogen (Rovetta et al., 2014). By blocking *Mtb*-Ag-induced IFN-γ, a marked reduction of autophagy was measured in monocytes from HR patients. In contrast, the incorporation of small quantities of IFN-γ significantly augmented autophagy in LR patients (Rovetta et al., 2014). We also demonstrated that IL-17A increased autophagy in infected monocytes from HR patients (Tateosian et al., 2017). However, in severe LR TB patients’ monocytes, a defect in the ERK1/2 signaling pathway prevented an augment in autophagy caused by IL-17A. Both IFN-γ and IL-17A increased the levels of autophagy in HR patients, promoting mycobacterial killing (Tateosian et al., 2017). Besides, Dang et al. demonstrated that addition of IL-26 to human *M. Leprae* infected monocytes induced autophagy (Dang et al., 2019). Furthermore, LC3-positive autophagosomes were mainly detected in lesions from T-lep (tuberculoid) as compared with L-lep (lepromatous) patients, indicating that *M*. *Leprae* dampened autophagy in human cells as an immune escape mechanism (Silva et al., 2017). It has been reported that type I glycoproteins such as SLAMF1 recruit molecules like Beclin-1 to the phagosome, participating in the connection to the cellular machinery that controls bacterial killing (Berger et al., 2010; Ma et al., 2012). Accordingly, we recently demonstrated that human neutrophils express SLAMF1 upon *Mtb*-stimulation, a protein that colocalized with LC3B^+^ vesicles (Pellegrini et al., 2020). Furthermore, SLAMF1 activation augmented neutrophil autophagy induced by *Mtb*, and neutrophils from TB patients showed reduced levels of SLAMF1 and lower amounts of autophagy against *Mtb* as compared to HD (Pellegrini et al., 2020). The eicosanoids, a family of potent biologically active lipid mediators, modulate immune responses in *Mtb* infection and have been suggested as potential HDT targets. Actually, manipulation of PGE2 and/or 5-LO was suggested to potentially counteract the type I IFN response in patients with severe TB as a HDT against *Mtb* (Mayer-Barber et al., 2014). Recently, we reported that PGE2 promotes autophagy in monocytes and neutrophils cultured with *Mtb*. We demonstrated that PGE2 augmented the percentage of LC3^+^ neutrophils and monocytes upon *Mtb*-Ag stimulation. Furthermore, the exogenous addition of this eicosanoid triggered a functional autophagy flux both in monocytes and lymphocytes from TB patients (Pellegrini et al., 2021). Thus, according to our results, PGE2 might be a new target for the development of novel therapeutic tools to fight *Mtb*. **Table 1** summarizes the data mentioned in this section.

### Non-Coding RNAs in Autophagy Modulation During Human Tuberculosis

In recent years, there was a growing body of evidence suggesting a critical role of non-coding RNAs (ncRNAs) in regulating host-pathogen interactions and immunity. A variety of pathogens, including *Mtb*, have been described to modulate the expression of these modulators by evading host responses and influencing the outcome of the infection (Staedel and Darfeuille, 2013; Zhang et al., 2019). Actually, some authors have proposed that miRNAs/lncRNAs regulation is an important strategy employed by *Mtb* to survive inside host cells (Kundu and Basu, 2021). Mycobacteria can alter the host miRNA expression profile for their benefit, affecting antimicrobial responses, cytokine production, metabolism and inflammation, among other processes (Yang and Ge, 2018). Moreover, the differential miRNA and lncRNA profiles detected in clinical samples from TB patients have led to an increasing interest in their use as TB biomarkers (Sabir et al., 2018). Importantly, some of these TB-associated ncRNAs play a role in the regulation of autophagy during *Mtb* infection, although most of these studies have been performed in murine models or cell lines.

Few reports have explored the role of these intermediaries in autophagy by using primary human cells from TB patients (**Table 2**). Accordingly, by analyzing GSE 29190 and GSE34608 miRNA microarray datasets Kim et al. detected that only 10 miRNA were differentially expressed in peripheral blood mononuclear cells (PBMCs) and biopsies from lungs and lymph nodes from TB patients, for example, miR-144* (Kim et al., 2017). Importantly, the authors demonstrated that miR-144* targets DRAM2 (an interactor of Beclin 1 and UVRAG) in human monocytes/macrophages, thus affecting autophagosome formation (Kim et al., 2017). Consequently, DRAM2 levels were decreased in monocytes from TB patients as compared to HD (Liu G. et al., 2020).

Furthermore, some studies have used primary cells obtained directly from the site of infection. Accordingly, Chen et al. demonstrated that miR-30A suppresses the elimination of intracellular *Mtb* by inhibiting autophagy. In fact, a higher concentration of miR-30A in alveolar macrophages from bronchoalveolar lavage (BAL) of smear-positive patients were detected in comparison with smear-negative patients and HD. Moreover, the expression of this miRNA decreased upon anti-TB treatment (Chen Z. et al., 2015). Besides, circAGFG1 was found to upregulate autophagy in *Mtb*–infected alveolar macrophages by targeting miRNA-1257, which in turn suppresses Notch signaling pathway (Shi et al., 2020).

Notably, Li et al. observed that PCED1B-AS1, a 410-bp lncRNA, is down-regulated in TB patients, which is accompanied by increased autophagy (Li et al., 2019). This function is carried out through binding with miR-155 to control its expression. This observation is concordant with a previous work demonstrating that *Mtb* can manipulate cellular miR-155 expression to regulate Atg3 levels, decreasing autophagosome formation in human dendritic cells (Etna et al., 2018). Finally, one study has explored the role of miR-889 and autophagy to maintain latent TB status (Chen et al., 2020). Chen et al. observed an increased miR-899 expression in latent TB individuals as compared to HD, which was significantly restored after anti-TB therapy. The authors identified the cytokine TWEAK as the target of miR-899, which inhibits autophagy and maintains mycobacterial survival in a human TB granuloma model (Chen et al., 2020). In summary, the increasing evidence found in murine models and cell lines demonstrates that some miRNAs/lncRNAs directly participate in the host response to *Mtb* by modulating autophagy. These studies were partially confirmed in human studies as we summarized here. Then, ncRNA-based therapeutics appear as an attractive target to directly modulate autophagy as novel HDT. However, an efficient drug design including ncRNA should be protected from degradation and successful delivery to the site of infection has to be ensured (Singh et al., 2021).

### Genetic Association Studies in Autophagy-Related Genes

Host and environmental factors have been shown to play a role in the pathogenesis and development of TB. For thousands of years, *Mtb* has co-evolved with humans, suggesting a powerful evolutionary pressure between the host and pathogen genomes, and therefore a strong impact of genetic factors on the development of different TB stages (Campbell and Tishkoff, 2008; Comas et al., 2013; Galagan, 2014). One particularly powerful approach to assess the role of some processes in humans is to investigate whether genetic variation influences susceptibility to infection. Single Nucleotide Polymorphisms (SNPs) are believed to be the true source of variability among humans (Pacheco and Moraes, 2009; Singh et al., 2017) and it is expected that the variants in genes involved in the pathogen-host interaction are influencing resistance/susceptibility to the disease.

Some reports have investigated SNPs in genes involved in the autophagy process, leading to important evidence about its role during human TB. For instance, IRGM1, a GTPase effector protein that plays an essential role in autophagy induction, has been studied in several populations. Rs9637876 IRGM1 SNP was associated with decreased susceptibility to TB caused only by *Mtb* Euro-American lineage in Ghana (Intemann et al., 2009). Moreover, the rs10065172 SNP within its coding region was associated with susceptibility to TB among African-Americans and in a Chinese population (King et al., 2011; Lu et al., 2016). Interestingly, this polymorphism was previously associated with mortality of patients with severe sepsis (Kimura et al., 2014). Furthermore, Yuan et al. identified three polymorphisms in the IRGM1 promoter region and found that CT genotype of rs4958846 decreased the risk of pulmonary TB in comparison with TT genotype (Yuan et al., 2016). In addition, Horne et al. selected ULK1 and GABARAP as candidate genes since they play fundamental roles in autophagy initiation and autophagosome maturation, respectively. Thus, they identified 2 SNPs in ULK1 (rs12297124 and rs7300908) in Asian participants that were significantly associated with latent TB. Moreover, ULK1-deficient cells had increased *Mtb* replication, decreased TNF response to stimulation, and impaired autophagy. Intriguingly, a previous work had investigated 22 polymorphisms of 14 autophagy genes in an Indonesian population. The authors found associations between SNPs in LAMP1 and MTOR genes and infection with *Mtb* Beijing genotype, but all those associations lost statistical significance after correction for multiple testing (Songane et al., 2012). Similarly, no associations were found in ATG5 (rs2245214, c.574-12777G>C) and NOD2 (rs2066844, c.2104C>T) genes in Romania (Cucu et al., 2017). Besides, 2 SNPs in the P2X7 gene coding for a plasma receptor that mediates ATP-induced autophagy, both in the promoter (Li et al., 2002) and the coding region (Fernando et al., 2007) were found to be associated with protection against TB. Finally, Cheng et al. found that rs1010 SNP in the VAMP8 gene is significantly associated with pulmonary TB in a Chinese Han population (Cheng et al., 2019). More comprehensive studies are required to evaluate the contribution of autophagy in different contexts because these studies are influenced by ethnicity, infection strains, polygenicity, among others. **Table 3** resumes the results cited in this section.

### Autophagy Activating Compounds for Human Host Directed Therapy in Tuberculosis

Autophagy modulation may signify a promising HDT strategy to fight human TB (Yuk et al., 2009). However, the clinical knowledge about HDT implementation is still widely deficient. HDT compounds combined with current TB drugs could shorten and improve treatments against *Mtb* infection. Therefore, autophagy activation by newborn drugs, soluble mediators or agents administered alone or in combination with anti-TB antibiotics still requires long-term clinical trials. Nevertheless, preclinical studies revealed that repurposing licensed drugs with a demonstrated potential to induce autophagy showed an effective therapeutic manipulation of host immunity against *Mtb* infection. These drugs displaying safe and pharmacokinetic profiles are promising for the evaluation of their effectiveness in randomized and controlled clinical trials. Accordingly, several clinical trials (clinicaltrilas.gov) have been conducted implementing dietary supplementation of the immunomodulator Vitamin D3. Innate immunity mediated by Vitamin D3 conferred protection against infection with *Mtb* (Yuk et al., 2009). Interestingly, Vitamin D3 and autophagy are physiologically linked *via* human cathelicidin (hCAP-18/LL-37), which activates transcription of autophagy-related genes such as Beclin-1 and Atg5 (Yuk et al., 2009). In the last ten years, numerous trials were performed supplementing Vitamin D3 as an adjunctive therapy. Nevertheless, differences in these trial outcomes have hampered the interpretation about Vitamin D3 efficacy as HDT for TB. Moreover, the impact of Vitamin D3 as an adjunctive therapy displayed no effect on culture conversion and sputum smear negativization (Ralph et al., 2013; Daley et al., 2015; Tukvadze et al., 2015; Ganmaa et al., 2017; Wu et al., 2018). However, genetic variation in the Vitamin D receptor gene was suggested to modify the effects of adjunctive Vitamin D3 in TB patients (Jolliffe et al., 2016). Additionally, multiple randomized trials suggested that adjunctive Vitamin D treatment has limited effect in improving clinical and immunologic outcomes during active *Mtb* infection despite evidence that specific VDR polymorphisms are predictive of sputum conversion time (Xia et al., 2014; Grobler et al., 2016; Zittermann et al., 2016). A phase 2 clinical study in TB patients (NCT02968927) assess the anti-inflammatory effects of Vitamin D3 in combination with 3 other adjunctive HDT compounds: CC-11050, Everolimus and Auranofin (Wallis et al., 2021). The CC-11050 is a type 4 phosphodiesterase inhibitor that displays anti-inflammatory properties (Subbian et al., 2016a; Subbian et al., 2016b); Everolimus, a serine/threonine-protein kinase mTOR inhibitor, is an autophagy inducer (Cerni et al., 2019) and Auranofin is an anti-inflammatory gold salt with antimicrobial activity against *Mtb* (Harbut et al., 2015). The preliminary results confirmed that CC-11050 and Everolimus are safe and well tolerated indicating a potential benefit to current TB treatment (Wallis et al., 2021). In other studies, Metformin, the AMPK-activating antidiabetic drug, was shown to inhibit the intracellular replication of *Mtb*, restrict disease immunopathology and enhance conventional anti-TB drug efficacy (Singhal et al., 2014). Moreover, in a pre-clinical study metformin administration in combination with either isoniazid (INH) or ethionamide (ETH) was reported to decrease *Mtb* load in lungs of infected mice (Singhal et al., 2014). Besides, the combined therapy including metformin with standard TB antibiotics was associated with beneficial consequences on clinical outcomes in TB (Singhal et al., 2014). Furthermore, an ongoing randomized clinical trial (NCT-CTRI/2018/01/011176) is evaluating the safety and efficacy of metformin as an adjunct used with rifampicin (RIF), INH, ETO and pyrazinamide (PZA) in patients with pulmonary TB (Padmapriyadarsini et al., 2019). Moreover, a new clinical trial in TB/HIV co-infected patients (Phase II A randomized, open-label trial, NCT04930744) is analyzing the effect of metformin with standard anti-TB drugs plus anti-retroviral therapy (Sullivan and Ben Amor, 2012; Marupuru et al., 2017).

Besides, statins (cholesterol-lowering drugs that inhibit β-hydroxy β-methylglutaryl-CoA (HMG-CoA) reductase) reduce the risk of coronary disorders and hypercholesterolemia. However, statins can also influence immunologic responses (Parihar et al., 2014). In pre-clinical models, statins such as Simvastatin, Rosuvastatin and Atorvastatin decreased *Mtb* load by enhancing autophagy, phagosomal maturation, and reducing pulmonary pathology, which suggests a potential role for statins as HDT in TB (Lobato et al., 2014; Parihar et al., 2014). Consequently, statins are among the most promising HDT agents for TB. The purpose of the numerous clinical trials that are currently undergoing is to assess the security, tolerance and pharmacokinetics of Pravastatin (NCT03882177) or Atorvastatin (NCT04147286) as adjunctive therapy when combined with standard TB drugs in adults infected with *Mtb*. There is still a long way to go by investigating many other repurposing licensed drugs with the ability to induce autophagy. For example, the mucokinetic Ambroxol (Choi et al., 2018), the antidiarrheal drug Loperamide (Lee, 2015; Juárez et al., 2016), the anti-protozoal drug Nitazoxanide (Lam et al., 2012), the anti-seizure drug Carbamazepine and Valproic acid (Schiebler et al., 2015), psychotropic or antidepressant drugs such as Nortriptyline, Fluoxetine and Prochlorperazine edisylate and Fluoxetine (Sundaramurthy et al., 2013; Stanley et al., 2014) are some of the drugs with potential use as HDT for TB treatment.

### Manipulating Autophagy to Improve Vaccination Against TB

The role of autophagy as a defense mechanism allows to hypothesize that vaccines that increase the autophagic response might be more effective in preventing the reactivation of latency or the acquisition of active TB. In fact, autophagy could be key in the development of effective TB vaccines since this process has the potential to improve the host immune response against *Mtb.* The attenuated *Mycobacterium bovis* Bacillus Calmette-Guérin (BCG) is effective in protecting against pulmonary and extrapulmonary TB in children up to 10 years old (Sterne et al., 1998; Abubakar et al., 2013), but protection against the pulmonary form of TB in adults remains highly controversial (Hatherill et al., 2020). BCG is able to affect the activation of T cells by evading phagosome maturation, autophagy, and by reducing MHC-II expression of antigen-presenting cells (APCs) (Russell, 2001). To avoid these deficiencies, an autophagy-inducing, TLR-2 activating C5 peptide from *Mtb*-derived CFP-10 protein was overexpressed in BCG in combination with Ag85B. This recombinant BCG was shown to induce stronger and longer-lasting immunity, increasing protection in a TB murine model (Khan et al., 2019). Furthermore, overexpression of Ag85B in BCG induced autophagy in APCs and increased immunogenicity in mice, indicating that vaccine efficacy can be augmented by enhancing autophagy-mediated antigen presentation (Jagannath et al., 2009). Therefore, exacerbation of autophagy could contribute to increase the immune response conferred by BCG. Interestingly, BCG was also genetically modified to improve its immunogenicity by replacing the urease C encoding gene with the listeriolysin encoding gene from *Listeria monocytogenes*. As a result, BCGΔureC::hly (VPM1002) was demonstrated to promote apoptosis and autophagy and facilitate the release of mycobacterial antigens into the cytosol (Nieuwenhuizen et al., 2017). The use of VPM1002 vaccine in preclinical trials has been shown to be more effective and safer than BCG (Nieuwenhuizen et al., 2017).

## Perspectives

For most countries, the end of TB as an epidemic disease and a public health problem still remains an aspiration rather than a reality. Current treatments still depend on antibiotic therapy and, considering the increasing antibiotic resistance, additional therapeutic targets are becoming progressively essential. To this end, the modulation of the autophagy process arises as an attractive goal. However, a deeper study of the cellular mechanisms that operate in humans are required, especially in TB patients where the infective status of each subject might have a special impact on autophagy modulation. As described here, at present the information regarding the human autophagic response during *Mtb* infection is very limited and precludes a better understanding of the process. In fact, the patients’ genetic background, among other factors, could be determinant in the development of their specific response to *Mtb* infection, influencing the effectiveness of a particular treatment. Furthermore, most of the existing studies are focused on autophagy in macrophages as the main target for *Mtb*, but this process is implicated in a wide variety of cell types. For example, autophagy has been shown to be critical during T cell activation and differentiation, central processes in TB immunity. Thus, an expanded analysis over the autophagy process in other cells such as lymphocytes, dendritic cells, neutrophils, basophils, among others would be necessary. Moreover, a broad examination of the immune responses of TB patients following an autophagy-modulating treatment would be extremely informative.

**Figure 1:**
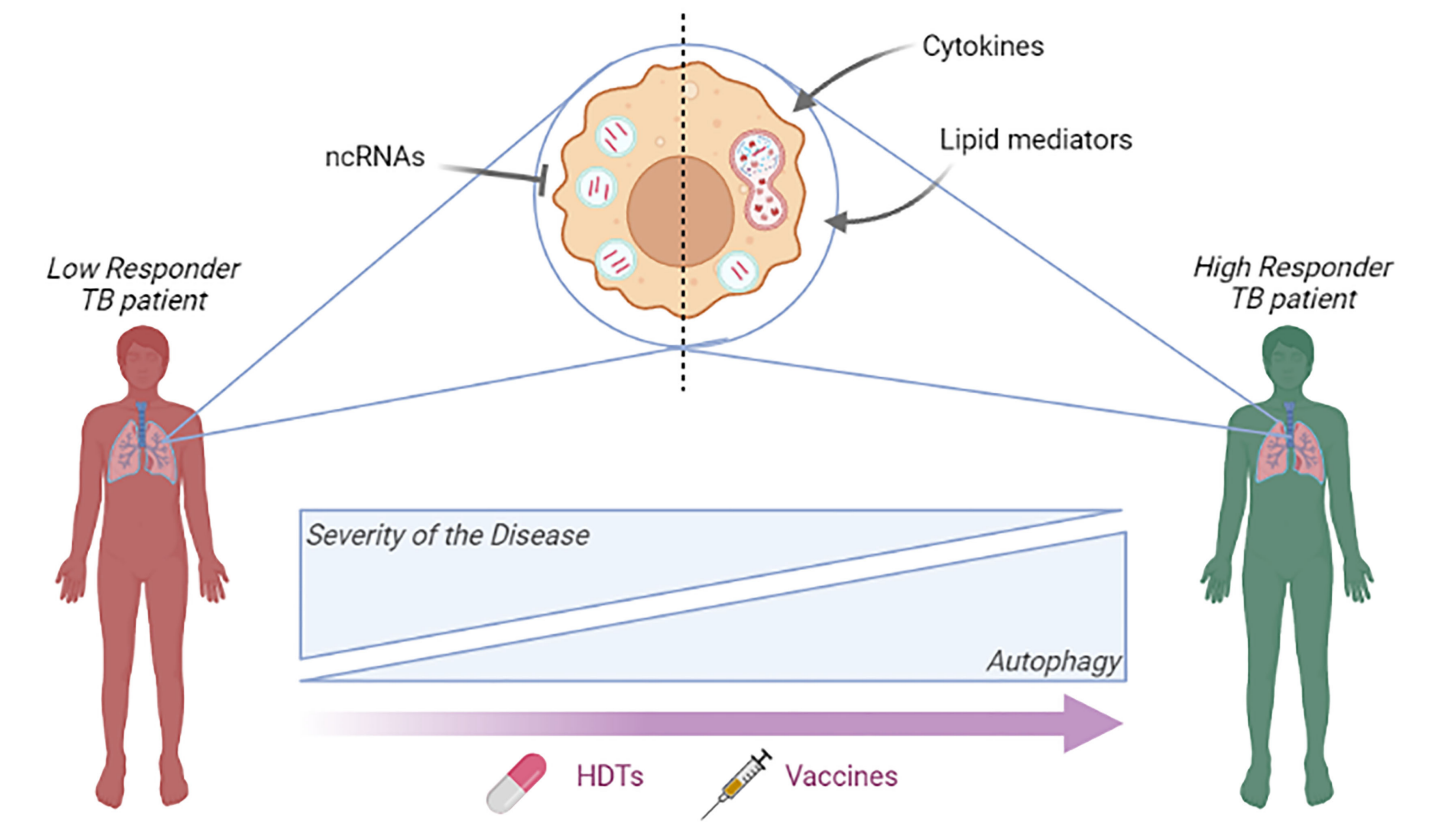
A simplified model of the role of autophagy in TB patients according to their immunological response to *Mtb*. The potential influence of immunological mediators and ncRNAs on autophagy is shown. High responder TB patients correspond to patients that display strong T cell immunity to *Mtb*, which correlates with milder manifestations of the disease (Pasquinelli et al., 2004; Jurado et al., 2012) and robust autophagic responses, as previously demonstrated (Rovetta et al., 2014; Tateosian et al., 2017). In contrast, patients with weak or no T cell responses to *Mtb* are associated with more severe disease (Pasquinelli et al., 2004; Jurado et al., 2012) and diminished autophagy (Rovetta et al., 2014; Tateosian et al., 2017). The purple arrow indicates the hypothetical impact of new HDT strategies (e.g.: autophagy as adjuvant therapy) in the treatment of TB patients and novel vaccines inducing an autophagic response. Figure created with BioRender.com..

Based on the mentioned findings reported by several authors and our studies, we proposed a schematic summary of the potential role of autophagy in TB patients according to their immunological response to *Mtb* (**Figure 1**). Briefly, immunological mediators such as cytokines, lipid mediators or ncRNAs, influence autophagy in TB patients with different immunological response to the bacteria. Implementing novel HDT strategies such as the modulation of autophagy as adjuvant therapy or novel vaccines might improve the treatment of TB patients. Current and future studies on autophagy-based therapeutic candidates may contribute to possible therapeutic/prevention improvements against TB, directly impacting the lives of millions of individuals infected with *Mtb*.

## Author Contributions

The writing-original draft preparation as well as the writing-review, and editing of the manuscript was in charge of JP, NT, MM, and VG. JP and VG were responsible for the conceptualization and organization of the present work. VG was in charge of supervision and project administration. All authors have read and agreed to the final version of the manuscript.

## Funding

This work was supported by grants from Agencia Nacional de Promoción Científica y Tecnológica (ANPCyT) [PICT-2017-1451 and PICT-2019-01617 to VG] and Universidad de Buenos Aires (UBACyT) [20020170100127BA to VG].

## Conflict of Interest

The authors declare that the research was conducted in the absence of any commercial or financial relationships that could be construed as a potential conflict of interest.

## Publisher’s Note

All claims expressed in this article are solely those of the authors and do not necessarily represent those of their affiliated organizations, or those of the publisher, the editors and the reviewers. Any product that may be evaluated in this article, or claim that may be made by its manufacturer, is not guaranteed or endorsed by the publisher.
